# Identification of uterine leiomyosarcoma-associated hub genes and immune cell infiltration pattern using weighted co-expression network analysis and CIBERSORT algorithm

**DOI:** 10.1186/s12957-021-02333-z

**Published:** 2021-07-28

**Authors:** Xiaoqing Shen, Zhujuan Yang, Songwei Feng, Yi Li

**Affiliations:** 1 Department of Gynecology and Obstetrics, The Second Affiliated Hospital of Soochow University, Suzhou, People’s Republic of China; 2 Department of Gynecology, The Affiliated Jiangsu Shengze Hospital of Nanjing Medical University and Jiangsu Shengze Hospital, 1399 Shunxin Middle Road, Suzhou, 215228 Jiangsu Province People’s Republic of China

**Keywords:** Uterine leiomyosarcoma, CIBERSORT, Weighted co-expression network, Neddylation

## Abstract

**Background:**

While large-scale genomic analyses symbolize a precious attempt to decipher the molecular foundation of uterine leiomyosarcoma (ULMS), bioinformatics results associated with the occurrence of ULMS based totally on WGCNA and CIBERSORT have not yet been reported. This study aimed to screen the hub genes and the immune cell infiltration pattern in ULMS by bioinformatics methods.

**Methods:**

Firstly, the GSE67463 dataset, including 25 ULMS tissues and 29 normal myometrium (NL) tissues, was downloaded from the public database. The differentially expressed genes (DEGs) were screened by the ‘limma’ package and hub modules were identified by weighted gene co-expression network analysis (WGCNA). Subsequently, gene function annotations were performed to investigate the biological role of the genes from the intersection of two groups (hub module and DEGs). The above genes were calculated in the protein–protein interaction (PPI) network to select the hub genes further. The hub genes were validated using external data (GSE764 and GSE68295). In addition, the differential immune cell infiltration between UL and ULMS tissues was investigated using the CIBERSORT algorithm. Finally, we used western blot to preliminarily detect the hub genes in cell lines.

**Results:**

WGCNA analysis revealed a green-yellow module possessed the highest correlation with ULMS, including 1063 genes. A total of 172 DEGs were selected by thresholds set in the ‘limma’ package. The above two groups of genes were intersected to obtain 72 genes for functional annotation analysis. Interestingly, it indicated that 72 genes were mainly involved in immune processes and the Neddylation pathway. We found a higher infiltration of five types of cells (memory B cells, M0-type macrophages, mast cells activated, M1-type macrophages, and T cells follicular helper) in ULMS tissues than NL tissues, while the infiltration of two types of cells (NK cells activated and mast cells resting) was lower than in NL tissues. In addition, a total of five genes (*KDR*, *CCL21*, *SELP*, *DPT*, and *DCN*) were identified as the hub genes. Internal and external validation demonstrated that the five genes were over-expressed in NL tissues compared with USML tissues. Finally, the correlation analysis results indicate that NK cells activated and mast cells activated positively correlated with the hub genes. However, M1-type macrophages had a negative correlation with the hub genes. Moreover, only the *DCN* may be associated with the Neddylation pathway.

**Conclusion:**

A series of evidence confirm that the five hub genes and the infiltration of seven types of immune cells are related to USML occurrence. These hub genes may affect the occurrence of USML through immune-related and Neddylation pathways, providing molecular evidence for the treatment of USML in the future.

**Supplementary Information:**

The online version contains supplementary material available at 10.1186/s12957-021-02333-z.

## Introduction

Uterine leiomyosarcoma (ULMS) is rare, with only 1% of all uterine malignancies [1]. Compared with other gynecological tumors, the etiology and pathogenesis of ULMS are not yet clear. ULMS is the most common histological subtype of uterine sarcoma originating in the smooth muscles of the myometrium. Because ULMS is not sensitive, treatment options are available, and it accounts for a considerable proportion of uterine cancer deaths [2]. The overall 5-year survival rate for ULMS is only 25% [3]. According to relevant guidelines [4], surgery is the primary treatment for ULMS [5–7]

Because the effect of adjuvant radiotherapy and chemotherapy on improving the survival of patients was only minimal, even with standard treatment, 50–71% of these patients would develop recurrence. Recently, the development of targeted therapy has been developed rapidly, and it is applied in lung cancer [8], ovarian cancer [9], and other malignant tumors [10, 11], which is expected to be an effective treatment for ULMS in the future. But ULMS has not been intensively investigated because they are given the few tumor intratumoral lymphocytes (TILs) on H&E sections and the low mutational burden [12]. Therefore, it is vital to explore the molecular etiology and immune-related pathogenesis of ULMS. It hopes that locate molecular targets and related pathways for treatment. In this regard, the current success of immune checkpoint blockade (ICB)-based cures in a variety of difficult-to-treat cancers raises the query of whether such cures would be applicable in ULMS. Several biomarkers indicating a potential for ICB have been pronounced in ULMS [13, 14]. The doable gain of ICB in ULMS was used to be illustrated by George et al. [15], who suggested the case of a patient who received anti-PD-1 monotherapy and experienced whole ailment remission for over 2 years.

With the development of sequencing, bioinformatics analysis plays an essential role in medical research. It has provided a somewhat objective basis for scientists’ exploration of tumor pathogenesis. Weighted gene co-expression network analysis (WGCNA) is a novel bioinformatics technique in which it can construct modules by analyzing gene expression profiles, and associate modules and sample characteristics [16]. Compared with statistics that solely focal point on differential expression, WGCNA has the following advantages: it can take full advantage of information, associate interesting alternations of phenotypes, and avoid the defects of differential expression evaluation artificially setting thresholds [17, 18]. The CIBERSORT algorithm can be used to assess the infiltration of immune cells in tissues, which has become a common technical approach in the field of immunology [19]. Newman et al. [20] present CIBERSORT method for the usage of RNA mixtures from almost any tissue and reveal it extensively increased accuracy for the evaluation of mixtures. Overall, CIBERSORT consistently outperformed other methods. Recently, abundant researches have used this algorithm to explore the function of immune cells in diseases, such as pancreatic cancer [21], small cell lung cancer [22], and endometrial carcinoma [23].

To the best of our knowledge, no research has focused on ULMS based on WGCNA and CIBERSORT in recent years. So, our study found hub genes and immune cells highly related to ULMS occurrence by analyzing USML datasets in the GEO database. It will fill the bioinformatics analysis gaps in ULMS and provide novel therapeutic ideas and research.

## Materials and methods

### Identification of differentially expressed genes

The datasets (GSE67463 as training set, GSE764, and GSE68295 as external validation set) were obtained from the Gene Expression Omnibus database (GEO, https://www.ncbi.nlm.nih.gov/) in NCBI based on the keywords: uterine leiomyosarcoma, and homo sapiens. The differentially expressed genes in ULMS samples and NL samples from the GSE67463 dataset were screened using the ‘limma’ package in R software. The thresholds in ‘limma’ package were set to |log2 fold change (FC)|> 2 and adjusted *P* value < 0.05.

### Co-expression network construction

Firstly, the outlier samples were identified by using a flash cluster package with a threshold setting of 80 and only 1 outlier sample was removed. The correlation coefficient between the two genes constituted the correlation matrix of co-expression. The above correlation matrix was constructed by the average linkage matrix and Pearson correlation method. Subsequently, the correlation matrix was transformed into an adjacency matrix in the formula amn =|cmn|β. The correlation coefficient was significant: the correlation coefficient of gene m and gene N is represented using amn, and the connection coefficient of gene m and gene N is represented by cmn. It was worth noting that β is a soft threshold (β = 9), making the strong association between genes more robust and vice versa. Finally, similar genes were put into the same module, and the adjacency matrix is transformed into a topological overlap matrix based on the above soft threshold. Pearson correlation analysis was carried out to evaluate the relationships between modules and ULMS occurrence.

### Functional enrichment analysis

Gene ontology (GO) and Kyoto Encyclopedia of Genes and Genomes (KEGG) analyses were performed in the genes from intersections of two groups (hub module and DEGs) by using related packages in R software.

### Identification of hub genes and construction of PPI network

In order to screen the genes from intersections of two groups (hub module and DEGs) in ULMS patients, the protein–protein interaction (PPI) network was constructed using the STRING tool Cytoscape software. Subsequently, according to the topological properties degree, the PPI networks above genes were calculated to select the hub genes further.

### Immune cell infiltration in ULMS tissues

CIBERSORT is a novel algorithm that mainly uses 547 immune cell-related gene expression values to estimate 22 immune cells in the tissue. The proportion of 22 immune cells in ULMS tissues was calculated using the CIBERSORT algorithm. Then, we used this algorithm to study the infiltration of immune cells between ULMS and NL. Finally, Pearson correlation analysis was used to calculate the correlation coefficient between immune cell infiltration and hub gene.

### Cell culture and western blotting

The following leiomyosarcoma cell lines, antibodies, and relevant experimental results required were provided from the Yi Li, Nanjing Medical University. SK-LMS-1 and MES-SA cell lines were cultured in Minimum Essential Media with 10% Fetal Bovine Serum. All cell lines were plated into T75 flasks and treated with MLN4924 (0 – 1.0 μM). Westerns were carried out as previously detailed [24].

### Statistical analysis

All statistical analyses were performed using the R software (v.3.6.3). An unpaired *t* test was used to compare the different tissues. Pearson correlation analysis was used to verify the correlation between hub genes and immune cell infiltration. *P* < 0.05 was considered statistically significant.

## Results

### Identification of differentially expressed genes involved in ULMS

**Fig. 1:**
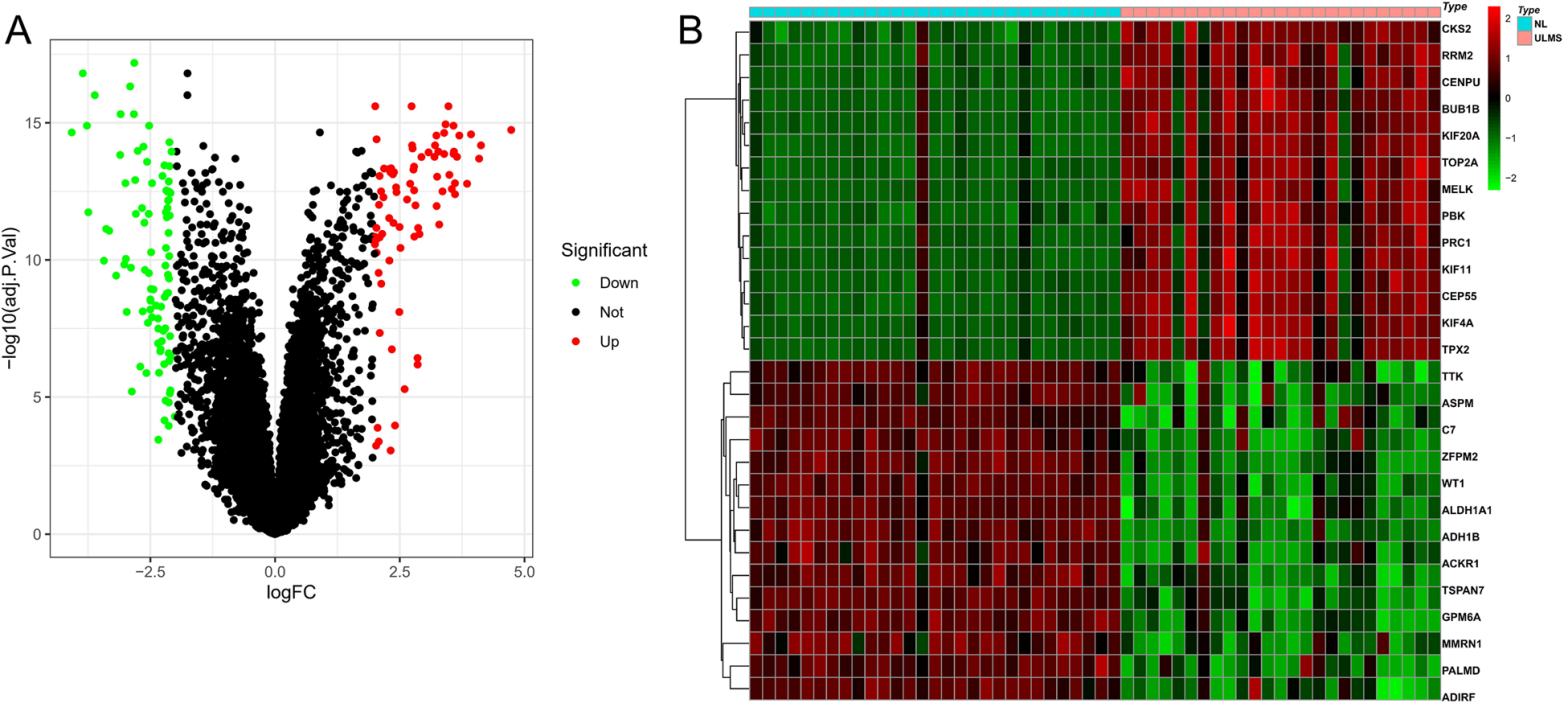
The expression of differentially expressed genes of ULMS and NL samples. **A** 171 DEGs were visualized by volcano plot and green and red indicated low and high expression in ULMS, respectively. In addition, black indicated that those genes no difference between ULMS and NL tissues. **B** DEGs were visualized by heatmap

According to the cut-off value determined in the “Materials and methods” section, 171 differential genes were finally identified in ULMS tissues and NL tissues. Volcano plot can discriminate ULMS and NL patients, in which the DEGs can be distinguished according to different colors, as shown in Fig. 1A. The red indicated the upregulated genes (79), and the green showed the downregulated genes (92), as shown in Table S1. In addition, black indicated that those genes no difference between ULMS and NL tissues. The heatmap showed the distribution of the top 30 DEGs in ULMS and NL, as shown in Fig. 1B.

Taken together, our data showed that 171 DEGs were identified in ULMS tissues and NL tissues.

### Construction of WGCNA and identification of hub modules

**Fig. 2:**
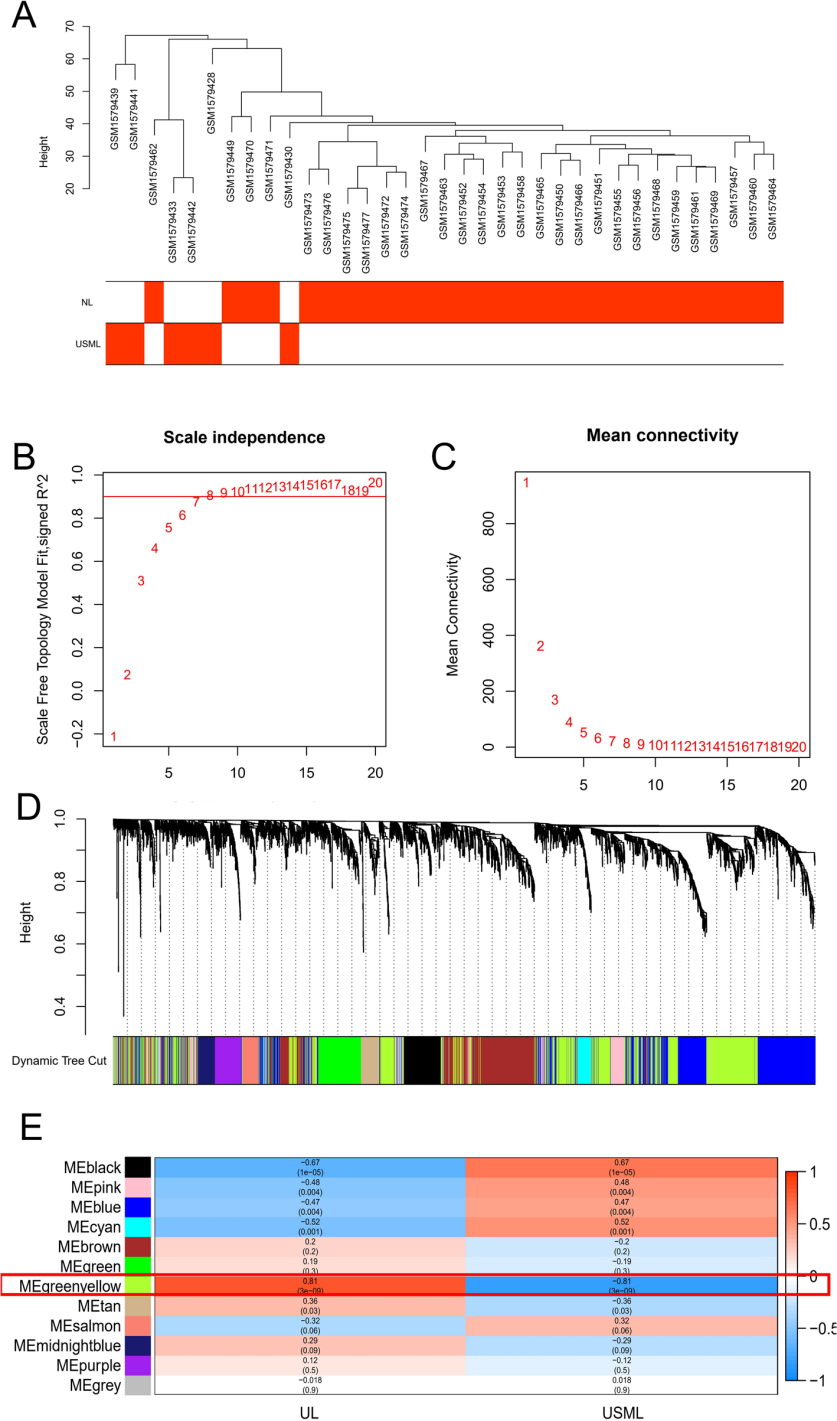
Construction of weighted gene co-expression network analysis. **A** Clustering dendrogram of samples in GSE67463 by cut-off = 80. ULMS samples were assigned as 1; NL samples were assigned as red. Color intensity is proportional to ULMS samples. **B** Different soft-threshold and corresponding scale-free topology model. **C** Different soft-thresholding powers and corresponding mean connectivity. **D** A part of genes with the same function in each module. **E** Correlation coefficient of each module with UL and USML

Fifty-four samples (25 ULMS and 29 NL) were clustered, and only one outlier sample was removed, as shown in Fig. 2A. In this way, the homogeneity of the remaining samples was improved, which is conducive to the accuracy of the results. According to the “Materials and methods” section, the correlation matrix was transformed into an adjacency matrix using the formula amn =|cmn|β (β = 9). In order to construct scale-free network distribution better, the “picks of threshold” function of the “WGCNA” package calculated the value of parameter β. Similar to scale-free network distribution, the correlation coefficient, mean connectivity, and average correlation between log (k) and log (P (k)) of each threshold (1–20) were calculated in ULMS and NL samples; if the average network connectivity corresponding to the threshold was close to 0, which indicates that the network connectivity is deficient, as shown in Fig. 2B, C. According to the corresponding steps of WGCNA modeling, a gene network was built based on a hierarchical clustering tree with the Diss Thres of 0.2. We took the minimum number of genes as 50 as the standard and used the dynamic pruning tree method to merge similar genes into each gene module. Finally, 12 modules are obtained, as shown in Fig. 2D. According to the thermogram of correlation between module and ULMS, the highest correlation coefficient between green-yellow module (1063 genes, 0.81/ − 0.81, *p* = 3e − 09), as shown in Fig. 2E.

Taken together, our data showed that a hub module (1063 genes) was identified in ULMS tissues and NL tissues.

### Functional enrichment analysis

**Fig. 3:**
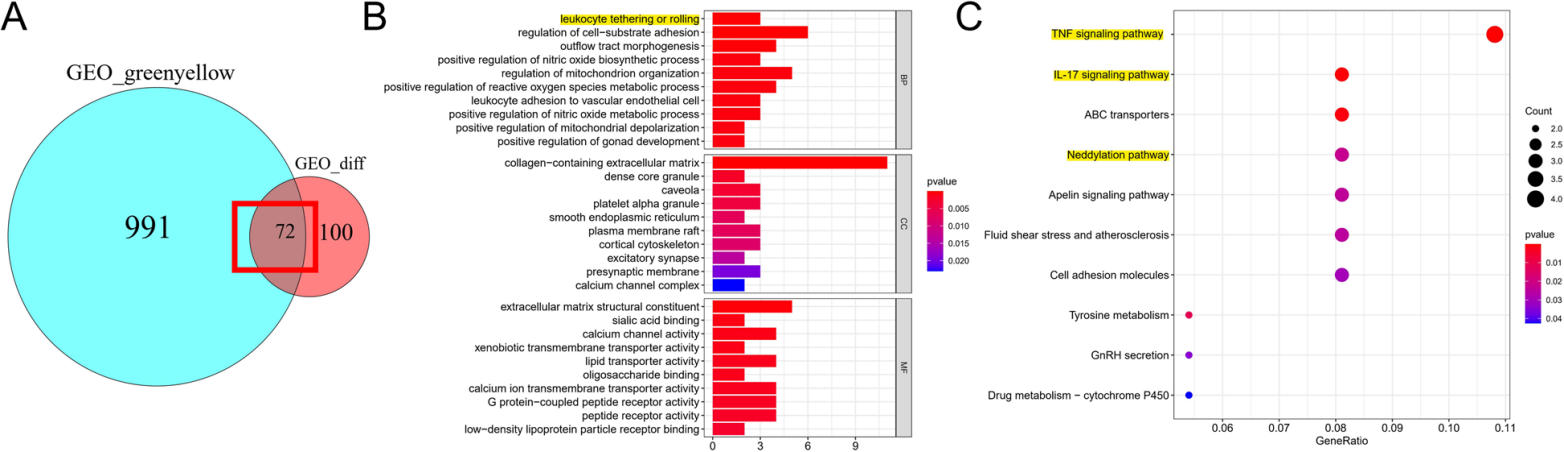
GO functional and KEGG pathway enrichment analysis. **A** 72 genes from DEGs and hub module. **B** Biological process (BP), cellular component (CC), and molecular function (MF) of GO enrichment. **C** KEGG pathway enrichment analysis. The size of the bubble indicates the strength of the *P* value

To further study the biological function of the genes in the hub module and DEGs, we screened out 72 genes from the gene intersection in two groups, as shown in the Venn plot (Fig. 3A). The 72 genes were then was included in KEGG and GO enrichment analysis. Interestingly, GO enrichment analysis showed that these genes mainly participated in the leukocyte tethering or rolling, as shown in Fig. 3B. In the meanwhile, KEGG enrichment analysis identified that these genes participated in the regulation of immune-related pathways and Neddylation pathway, as shown in Fig. 3C. This result may be revealed between ULMS and immunization, so we analyzed it in depth in the “Immune cell infiltration analysis” section and “Correlation between hub genes and Neddylation pathway” section.

Taken together, our data showed that an exciting possibility: ULMS related to immunization cell infiltration and the Neddylation pathway.

### Immune cell infiltration analysis

**Fig. 4:**
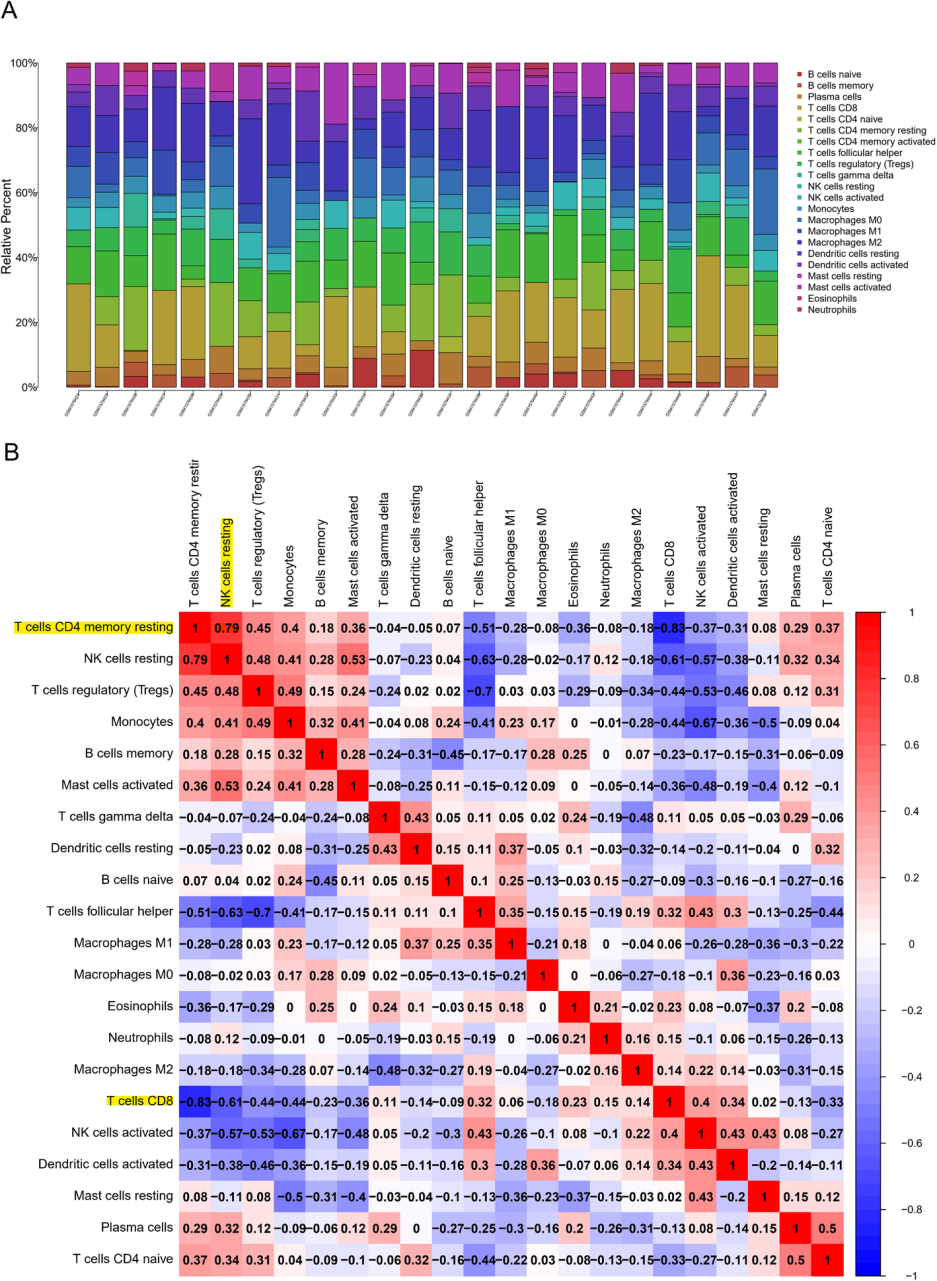
Immune cell infiltration pattern in USML tissues. **A** Proportion of the 22 immune cell types in USML tissues. **B** Correlation matrix between the 22 immune cell types

**Fig. 5:**
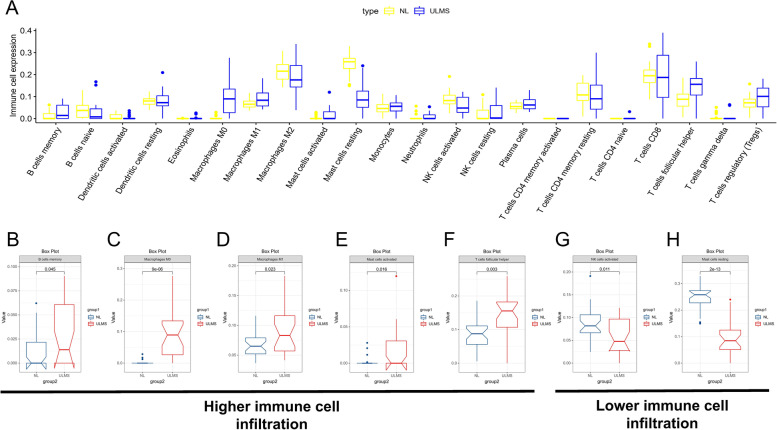
Different distribution of immune cell in NL and USML tissues. **A** The total distribution of immune cells in NL and USML tissues. **B** Memory B cells distribution. **C** M0-type macrophages distribution. **D** Mast cells activated distribution. **E** M1-type macrophages cell distribution. **F** T cells follicular helper cell distribution. **G** NK cells activated cell distribution. **H** Mast cells resting cell distribution

CIBERSORT algorithm was used to analyze the immune infiltration of ULMS samples. The proportion of 22 immune cells was shown in a bar plot, and macrophages account for most significant proportion among the immune cells in the samples, as shown in Fig. 4A. T cells CD4 memory resting had the strongest positive correlation with NK cells resting (0.79); however, T cells CD8 had the strongest negative correlation with T cells CD4 memory resting (− 0.83), as shown in Fig. 4B. In order to further compare the difference in proportion among the immune cells between NL and ULMS tissues, we also performed immune cell infiltration analysis in NL tissues, as shown in Fig. 5A. We found a higher infiltration of five types of cells (memory B cells, M0-type macrophages, mast cells activated, M1-type macrophages, and T cells follicular helper) in ULMS tissues than in NL tissues, while the infiltration of two types of cells (NK cells activated and mast cells resting) was lower than in NL tissues (Fig. 5B–H; *p* < 0.05).

Taken together, our data showed that the distribution of 22 type immune cells in USML and NL tissues, including seven types of cells related to USML occurrence.

### Identification of the hub genes

**Fig. 6:**
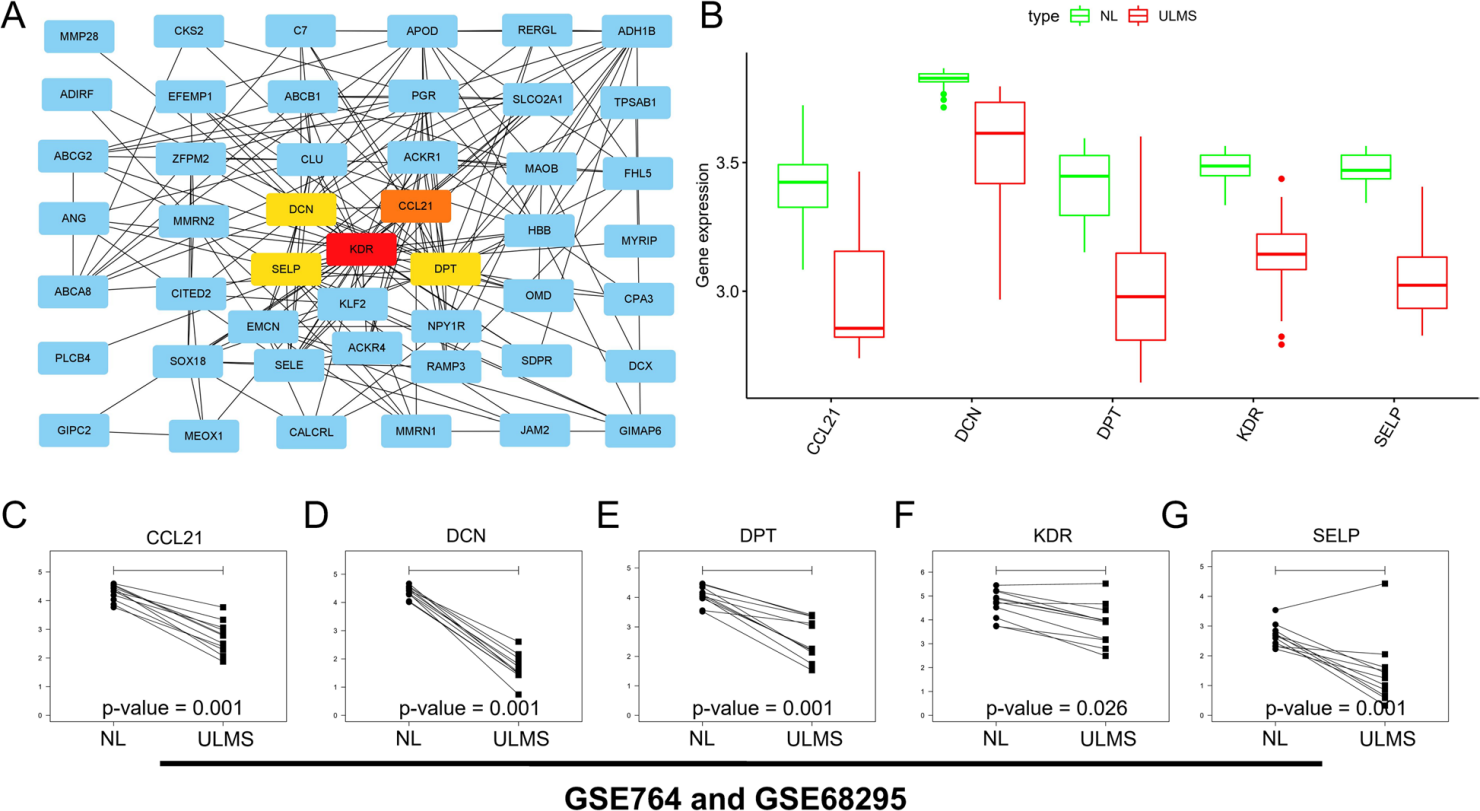
Internal and external validation for hub genes. **A** Identification of the hub genes from PPI network. **B** Heatmap of the hub genes. **C***CCL21* in external validation. **D***DCN* in external validation. **E***DPT* in external validation. **F***KDR* in external validation. **G***SELP* in external validation

The STRING online tool was used to construct a PPI network from the intersection of two groups (hub module and DEGs) with the node pair combing score ≥ 0.15 as the criterion, excluding disconnected nodes in the network. To further explore the hub genes, the data of PPI networks in STRING were input into the Cytoscape software. The topological properties analysis with top 5 of degree set as the criterion and five hub genes were screened. These nodes were *KDR* (degree = 28), *CCL21* (degree = 19), *SELP* (degree = 18), *DPT* (degree = 18), and *DCN* (degree = 18), as shown in Fig. 6A.

Taken together, our data showed that five hub genes (*KDR, CCL21, SELP, DPT, *and *DCN)* related to USML occurrence were identified.

### Internal and external validation for hub genes

Based on the hub genes, we have got *KDR*, *CCL21*, *SELP*, *DPT*, and *DCN*. We found that the hub genes were lower expressed in USML tissues than NL tissues by differential analysis based on the GSE67463 dataset, as shown in Fig. 6B. To verify the accuracy of the predicted results, hub genes expression in 11 pairs of USML and NL tissues was detected using external datasets (GSE764 including three pairs and GSE68295 including eight pairs). The results showed that the hub genes were over-expressed in NL tissues and consistent with the prediction results (Fig. 6C − G). The association of hub genes with different types of immune cell infiltration was explored. The results of Pearson correlation analysis indicate that NK cells activated and mast cells activated had a positive correlation with the hub genes. However, M1-type macrophages had a negative correlation with the hub genes, as shown in Table 1. PD-L1 has been correlated with immune response and is currently used as a biomarker for ICB therapy in ULMS, so we investigated the correlation of hub genes with PD-L1 and found that except *DCN*, other hub genes were significantly correlated with PD-L1, as shown in Figure S1.

**Table 1:** The correlation between hub genes and immune cell infiltration

Gene	Immune cell	*P* value	Correlation
*KDR*	Mast cells activated	0.026	Positive
*CCL21*	Mast cells activated	0.037	Positive
*SELP*	Mast cells activated	0.003	Positive
*DPT*	Mast cells activated	0.010	Positive
*DCN*	Mast cells activated	0.026	Positive
*KDR*	NK cells activated	0.029	Positive
*CCL21*	NK cells activated	0.002	Positive
*SELP*	NK cells activated	0.002	Positive
*DPT*	NK cells activated	0.009	Negative
*DCN*	NK cells activated	0.030	Negative
*KDR*	M1-type macrophages	0.001	Negative
*CCL21*	M1-type macrophages	0.001	Negative
*SELP*	M1-type macrophages	0.027	Negative

Taken together, our data showed that hub genes were lower expressed in USML tissues.

### Correlation between hub genes and Neddylation pathway

**Fig. 7:**
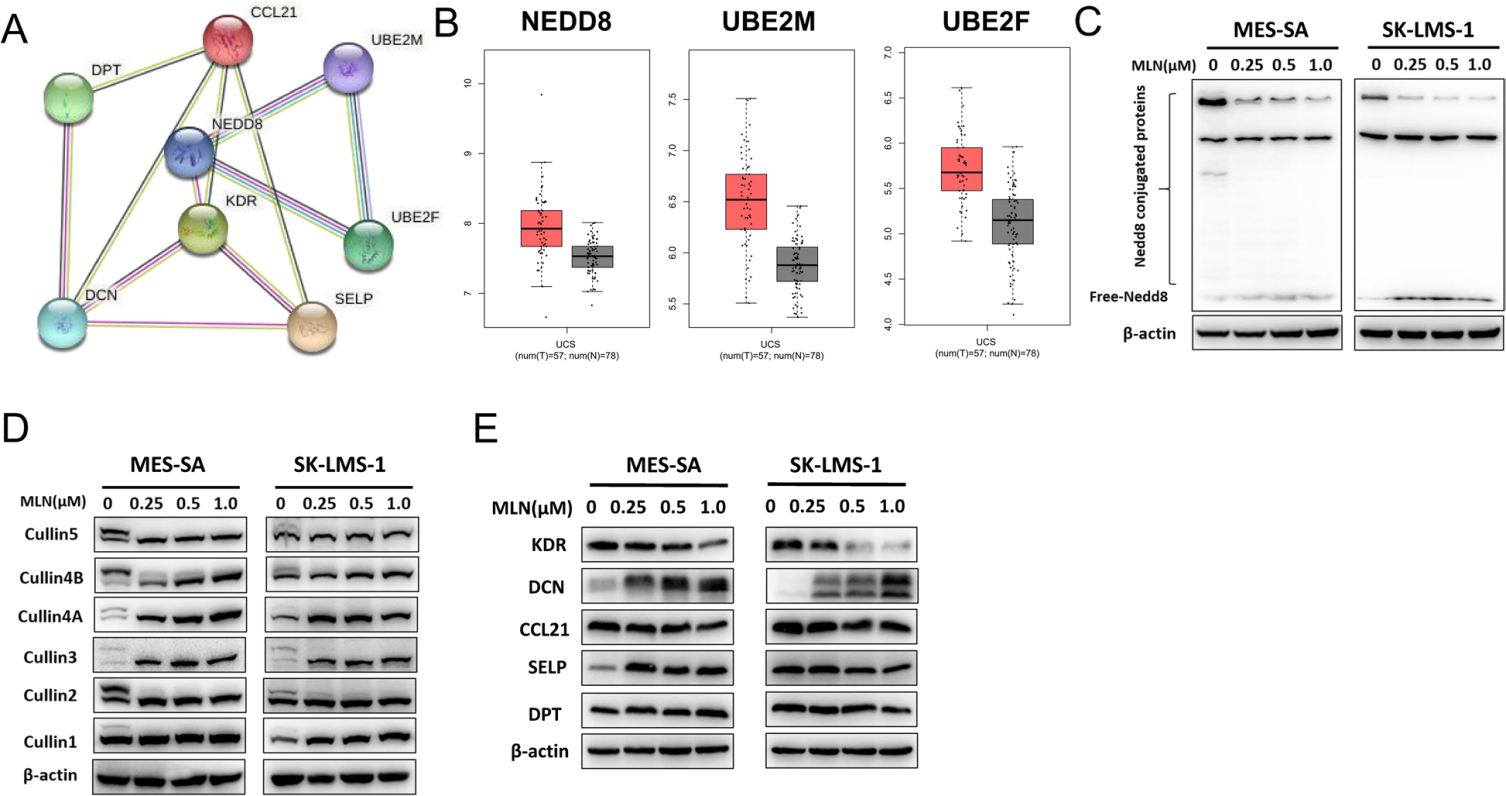
Correlation between hub genes and Neddylation pathway. **A** PPI network in hub genes and Neddylation-associated genes. **B***NEDD8*, *UBE2M*, and *UBE2F* expression in TCGA database. **C** Total protein Neddylation in MES-SA and SK-LMS-1. **D** Cullin protein Neddylation in MES-SA and SK-LMS-1. **E** Western blotting in MES-SA and SK-LMS-1 cell lines

In our and others’ previous study [25–28], Neddylation inhibitor MLN4924 has significant anti-tumor effect in both vitro and vivo. Through TCGA database and KEGG analysis, we found that NEED8, an important molecule in the Neddylation pathway, as well as the catalytic enzymes UBE2M and UBE2F, were all transcribed at higher levels in USML tissues than NL tissues (Fig. 3C and Fig. 7B). It indicated that the Neddylation pathway is activated in USML. It was worth noting that PPI analysis suggested an interaction between hub genes and Neddylation-related genes, as shown in Fig. 7A. Therefore, we made a bold conjecture: the Neddylation pathway may regulate hub genes (*KDR*, *CCL21*, *SELP*, *DPT*, and *DCN*). We detected the expression level of the hub gene in USML cell lines (MES-SA and SK-LMS-1). The results showed that the expression levels of *DCN* in two cells were significantly upregulated with the increase of MLN4924 concentration, while the other protein levels were almost unchanged, as shown in Fig. 7E. Moreover, immunoimprinting analysis was used to analyze the effects of different concentrations of MLN4924 on the Neddylation pathway in USML cell lines, including the Neddylation level of the total protein and the Neddylation level of the substrate Cullin protein. The results showed that MLN4924 significantly inhibited the Neddylation pathway of MES-SA and SK-LMS-1 cells, as shown in Fig. 7C–D.

Taken together, our data confirmed that the possibility of activation of Neddylation pathway in KEGG analysis (Fig. 3C) and hub gene (*DCN*) associated with Neddylation pathway.

## Discussion

Uterine sarcomas are sporadic mesenchymal neoplasms, and its related research is less than other malignant tumors. Because of the lower incidence rate, different histological appearances and clinical manifestations lead to no superior therapeutic regimen and lack of specific molecular markers [29]. Traditionally, the classification of uterine sarcomas is based on histological appearance, and immunohistochemistry (IHC) is chosen to support tissue differentiation. The most common subtypes of uterine sarcoma are leiomyosarcoma (ULMS), low-grade endometrial stromal sarcoma, and high-grade endometrial stromal sarcoma [30]. The behavior of ULMS is unpredictable. Even if the tumor is confined to the uterine body, recurrence and metastasis are very common [31]. Interestingly, related case has been reported successful pregnancy after complete resection of leiomyomatosis peritonealis without recurrence [32]. A better understanding of the biology of ULMS through clinically molecular markers will help to judge prognosis and treatment [33].

In the present study, based on the profiles, including GSE764, GSE64763, and GSE68312 from the GEO database, 171 DEGs were identified by comparing ULMS samples with NL samples. Hub modules related to ULMS were identified by weighted gene co-expression network analysis (WGCNA). The above two groups of genes were intersected to obtain 72 genes for subsequent functional annotation analysis and PPI analysis. Go and KEGG function enrichment analysis was performed in R package. This method can predict the function and pathway of related genes. Go functional annotation showed that the hub gene mainly participated in immune response, such as leukocyte rolling and adhesion. KEGG enrichment analysis showed that the above genes participated in regulating immune-related pathways and the Neddylation pathway. However, there is no relevant experimental evidence to prove that these pathways are related to the occurrence of ULMS. But Andre Pinto found that PD-L1 is expressed by the majority of carcinosarcomas, predominantly in the epithelial components [34]. Elisheva D showed that leiomyosarcomas demonstrate significantly higher PD-L1 expression and cytotoxic T cell infiltration when compared with other uterine smooth muscle tumors [14]. PD-L1 mediates multiple Immune-related pathways. Thus, it is reasonable to suggest that regulation of the immune system is closely related to the occurrence of USML. Although the pathways in ULMS have not been adequately discussed, the immune landscape and genomic landscape of ULMS have been reported [33, 35, 36]. Gotoh O revealed that POLE and MSI (hypermutator) tumors showed an enrichment of M1 macrophages, plasma cells, and CD8 + T cells, whereas CNH and CNL (non-hypermutator) tumors had high levels of M2 macrophages from gynecologic carcinosarcoma RNA-seq data [37]. This is similar to our results; we found a higher infiltration of five types of cells (memory B cells, M0-type macrophages, mast cells activated, M1-type macrophages, and T cells follicular helper) in ULMS tissues than in NL tissues, while the infiltration of two type of cells (NK cells activated and mast cells resting) was lower in NL tissues. In addition, the results of Pearson correlation analysis indicate that NK cells activated and mast cells activated had a positive correlation with the hub genes. However, M1-type macrophages had a negative correlation with the hub genes.

The topological properties analysis in PPI network screened out five hub genes: *KDR, CCL21, SELP, DPT*, and *DCN.* Current research focused on Kinase insert domain receptor (*KDR*) in infertility field, and the role of ULMS needs to be explored in the future. Chen found that increased KDR was found in the endometrium of intrauterine adhesions (IUA) patients, which was positively related to IUA severity [38]. *CCL21* promotes immune activity in the tumor microenvironment (TME) by colocalizing dendritic cells (DC) and T cells programming ectopic lymph node architectural structures that correlate with cancer prognosis [39]. CCL21 plays a role not only in immunity, but also in regulating the biological processes of tumor cells. Yang showed that CCL21 can suppress the migration and invasion of colorectal cancer cell line [40]. *SELP* (P-selectin) may contribute to adverse platelet function [41]. *DCN*, a small leucine-rich proteoglycan, is a tumor suppressor in prostate cancer [42]. Reduced expression of *DCN* has been considered as an indicator of poor prognosis in patients with cancer [43, 44]. According to the current literature, there is only indirect evidence to prove the accuracy of our prediction of hub genes associated with ULMS occurrence. However, the relationship between the hub genes and different immune cell infiltrates suggests they are correct. In particular, our subsequent western blotting further verified our conjecture.

Neddylation pathway is a novel protein post-translational modification. Studies have shown that Neddylation pathway is over-activated in a variety of human primary tumor tissues [45]. It can promote the development of tumor by activating CRLs (Cullin-Ring ligases) to cause the degradation of CRL tumor suppressor protein substrates. Neddylation inhibitor MLN4924 [46] has significant anti-tumor effect in both vitro and vivo. Through TCGA database analysis, we found that NEED8, an important molecule in the Neddylation pathway, as well as the catalytic enzymes UBE2M and UBE2F, were all transcribed at higher levels in USML tissues than NL tissues. It indicated that the Neddylation pathway is activated in USML. It was worth paying attention to what PPI analysis suggested that there was an interaction between hub genes and Neddylation-related genes. Therefore, we made a bold conjecture: hub genes (*KDR*, *CCL21*, *SELP*, *DPT*, and *DCN*) may be regulated by the Neddylation pathway. But only the *DCN* may be associated with Neddylation pathway.

The limitations of this research need to be discussed. There is no experimental evidence to prove the correlation between gene-related immune pathways and ULMS. Firstly, this research is in the prediction stage. Although some experiments have been carried out to verify the hub gene, there is no further evidence to verify our prediction results. Our follow-up studies are focusing on the detailed correlation between *DCN* gene and Neddylation pathway. We believe that our verification in vitro and in vivo will make the conclusion more rigorous in the future. Secondly, the sample size of the research is not large enough, which is also related to the low incidence rate of USML. Finally, due to the specificity of blood, we cannot guarantee the accuracy of CIBERSORT algorithm.

## Conclusions

A series of evidences confirm that the five hub genes and the infiltration of seven types of immune cells related to USML occurrence. These hub genes may affect the occurrence of USML through immune-related and Neddylation pathways, providing molecular evidence for the treatment of USML in the future.

## Supplementary Information

**Additional file 1: Figure S1**. Correlation between hub genes and PDL1. (A) The correlation between CCL21 and PDL1 (D) The correlation between DCN and PDL1 (E) The correlation between DPT and PDL1 (F) The correlation between KDR and PDL1(G) The correlation between SELP and PDL1.

**Additional file 2: Table S1**. The differentially expressed genes (DEGs) in NL and ULMS.

## Data Availability

The following information was supplied regarding data availability. Data is available at the Gene Expression Omnibus (GEO) database.
