# Supplementary figures and images for: Identification of uterine leiomyosarcoma-associated hub genes and immune cell infiltration pattern using weighted co-expression network analysis and CIBERSORT algorithm
Source: World J Surg Oncol. 2021 Jul 28;19:223. doi: 10.1186/s12957-021-02333-z (PMC8320213; doi:10.1186/s12957-021-02333-z)

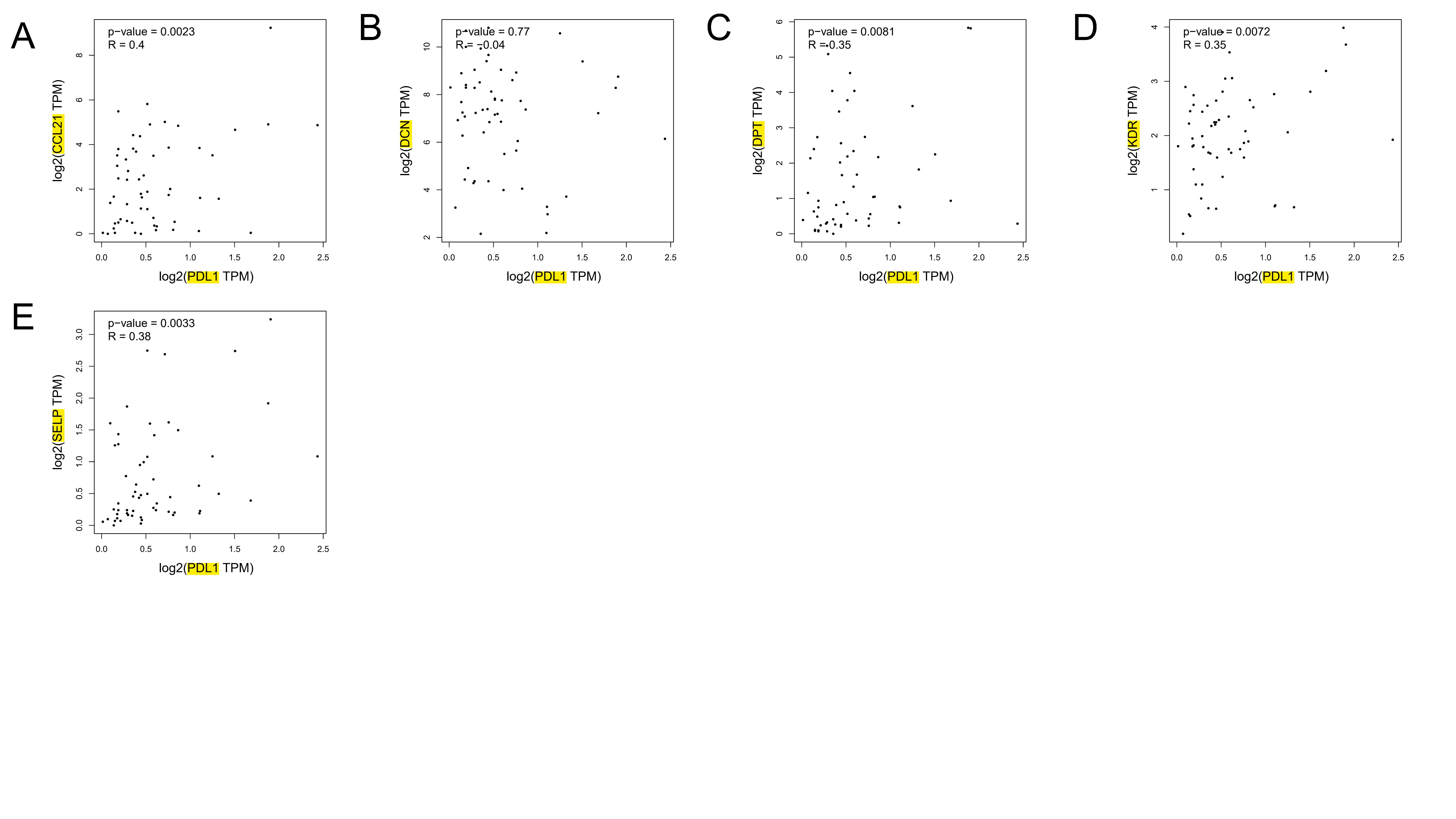

Supplement: Supplementary file 1 — Additional file 1: Figure S1. Correlation between hub genes and PDL1. (A) The correlation between CCL21 and PDL1 (D) The correlation between DCN and PDL1 (E) The correlation between DPT and PDL1 (F) The correlation between KDR and PDL1(G) The correlation between SELP and PDL1. [file 12957_2021_2333_MOESM1_ESM.png]
